# The focal adhesion protein Hic-5 is highly expressed in the rat myometrium during late pregnancy and labour and co-localizes with FAK

**DOI:** 10.1186/1477-7827-5-22

**Published:** 2007-06-05

**Authors:** Jenn M Croke, Luke RG Pike, Daniel J MacPhee

**Affiliations:** 1 Division of Basic Medical Sciences, Health Sciences Centre, Faculty of Medicine, Memorial University of Newfoundland, St. John's, NL, A1B 3V6, Canada

## Abstract

**Background:**

Myometrial growth and remodeling of the cytoskeleton and focal adhesions during late pregnancy may be critical aspects of myometrial activation and thus labour. Yet our understanding of these aspects is inhibited by the paucity of information concerning the components of focal adhesions in the myometrium. The focal adhesion protein hydrogen peroxide-inducible clone-5 (Hic-5) has recently been found in mononuclear smooth muscle but was not examined in the myometrium during pregnancy. Thus, the goal of this study was to characterize Hic-5 mRNA and protein expression in the rat myometrium during pregnancy and labour.

**Methods:**

Rat myometrium samples were obtained from non-pregnant animals, pregnant animals on days (d) 6, 12, 15, 17, 19, 21, 22, 23 (active labour) and 1 day postpartum (PP). In addition, myometrium samples were collected from rats within a progesterone-delayed labour paradigm. Hic-5 mRNA expression was analyzed by Northern blot analysis while Hic-5 protein expression was examined by immunoblot and immunofluorescence analysis.

**Results:**

Hic-5 mRNA expression on d15, d19 and d21 was found to be significantly elevated compared to d6 and d12 of pregnancy and expression on d23 was significantly elevated over d6 (p < 0.05). Immunofluorescence analysis demonstrated that detection of Hic-5 protein in the circular muscle layer appeared to increase from d17 onwards, except PP, and Hic-5 was detectable in the cell cytoplasm and more continuously associated with myometrial cell membranes. In the longitudinal muscle layer Hic-5 was readily detectable by d15 and thereafter and primarily associated at myometrial cell membranes. Co-immunofluorescence analysis of potential Hic-5 and focal adhesion kinase (FAK) association in situ demonstrated a limited level of co-localization on d19, d23 and PP in the circular muscle layer while in the longitudinal muscle layer Hic-5 and FAK were readily co-localized at myometrial cell membranes.

**Conclusion:**

Hic-5 is highly expressed in the rat myometrium during late pregnancy and labour and co-localizes with FAK in situ. Our results are consistent with a potential role for Hic-5 in focal adhesion remodeling in the rat myometrium during late pregnancy.

## Background

The initiation of labour contractions results from the complex interaction of maternal and fetal components [1,2]. During late pregnancy, the uterus undergoes a modification under the influence of mechanical signals (uterine stretch) and endocrine or paracrine signals of maternal and fetal origins [2]. The end result is a collective alteration in the uterine smooth muscle or myometrium, termed myometrial activation that is marked at the molecular level by the increased expression of a group of genes encoding contraction-associated proteins (CAPs) such as gap junctions and oxytocin receptors [reviewed in [2,3]]. As a result of myometrial activation, at term the uterine musculature is responsive to uterotonins, spontaneously active, and excitable.

It is now appreciated that myometrial growth and remodeling of the cytoskeleton and cell-extracellular matrix (ECM) contacts or focal adhesions during late pregnancy may be a critical aspect of myometrial activation and thus labour. During mid to late pregnancy in the rat, circulating levels of progesterone and uterine distension induced by growing fetuses leads to hypertrophic growth of the myometrium and significant myometrial tissue remodeling [4-9]. Specifically in the rat, expression of type IV collagen, laminin, and fibronectin are markedly increased and the proteins deposited around myometrial cells during late pregnancy [7,10]. Such tissue remodeling necessitates the reorganization of focal adhesions during late pregnancy to properly anchor growing myometrial cells to their ECM. Focal adhesions develop as a result of ligand-induced clustering of cell surface ECM receptors named integrins. They can mediate tension transmission between the contractile apparatus of the cell and ECM and also associate with various adapter proteins, kinases or growth factor receptors to connect them to the actin cytoskeleton and/or trigger biochemical signaling pathways [11,12]. A regulator of focal adhesion reorganization named focal adhesion kinase (FAK) was reported to be highly expressed and activated in the rat myometrium during late pregnancy and a FAK-binding adapter protein termed paxillin, which is involved in focal adhesion formation [13], was also highly tyrosine phosphorylated during this period [14]. We have also recently demonstrated that α5 integrin expression is elevated in the rat myometrium during late pregnancy and labour and may facilitate myometrial cell cohesion [15]. Furthermore, Shynlova and colleagues [8] have shown that γ-actin expression in the rat is markedly upregulated during mid and late pregnancy. Specifically, detection of γ-actin by immunohistochemistry demonstrated that during late pregnancy γ-actin became concentrated close to the cell membrane as a continuous ring. The authors have suggested that upregulation of γ-actin expression may contribute to myometrial hypertrophy and is likely a marker of the differentiated state of the tissue at this time.

Recently, Kuo and Seow [16] demonstrated that appropriate cytoskeletal filament organization, focal adhesion formation, and cell-cell and cell-ECM interactions were necessary in airway smooth muscle cells within a tissue bundle to function as a *mechanical syncytium *during contraction. We have since suggested that such a syncytium model of contraction may be relevant in the myometrium, as a component of the activation process, to facilitate efficient force transduction of the sustained, coordinated and powerful contractions of labour [15]. A stumbling block to determining whether a mechanical syncytium indeed develops in the myometrium during late pregnancy is the paucity of information concerning the components of focal adhesions and the molecular mechanisms of integrin-mediated signaling in the rat myometrium during late pregnancy and labour.

A focal adhesion protein that appears important for integrin-mediated signaling is hydrogen peroxide inducible clone-5 (Hic-5). Hic-5 is a paxillin homologue that was originally discovered as a transforming growth factor β1 (TGF β1) and H_2_O_2_-inducible gene by differential hybridization [17]. Analogous to paxillin, Hic-5 is localized to focal adhesions via its C – terminal LIM (derived from the founders LIN-11, Isl1, MEC-3) 2 and LIM 3 domains and also binds FAK likely via a leucine rich domain (LD) 3 in the N – terminus [18-20]. Furthermore, Hic-5 has been shown to compete with paxillin for FAK binding and control cell spreading thus providing a novel mechanism for regulation of integrin-mediated signaling [20]. Recently, it was reported that Hic-5 expression, unlike paxillin, was primarily found in mononuclear smooth muscle including the non-pregnant mouse and human myometrium [21,22] and transient over-expression of Hic-5 in SVS30 smooth muscle cells, embedded in 3D collagen gels, suppressed their contractile capability [21]. Based on such reports, the goal of this study was to characterize Hic-5 mRNA and protein expression in the rat myometrium during pregnancy in order to begin assessing its potential role in integrin-mediated signaling and the development of a mechanical syncytium in this smooth muscle tissue.

## Methods

### Animals

Sprague-Dawley rats were obtained from the Mount Scio Vivarium (Memorial University of Newfoundland, St. John's, Newfoundland, Canada). Animals were individually housed and cared for under standard environmental conditions (12 hour light and 12 hour dark) in the Animal Care Unit at the Health Sciences Centre, Memorial University of Newfoundland. Rats were fed LabDiet Prolab RMH 3000 (PMI Nutrition International, Brentwood, Missouri, USA) and water *ad libitum*. The institutional animal care committee approved all experiments under animal care protocols 02-02-DM – 02-04-DM. Virgin female rats (~220 g each) were mated with stud males and observation of vaginal plugs the following morning was designated day 1 post coitum. The time of delivery under these standard conditions was day 23 of gestation.

### Experimental design

#### Normal pregnancy and term labour

Individual animals were each placed in a euthanasia chamber and exposed to an increasing concentration of carbon dioxide gas resulting in death within 5–10 minutes. Tissues were collected at the following timepoints: non-pregnant (NP), gestation days (d) 6, 12, 15, 17, 19, 21, 22, 23 (labour) and 1 day postpartum (PP). Labour samples were taken during active labour and only after the rat had delivered two to three pups.

#### Progesterone-delayed labour

The onset of labour in the rat on d23 of gestation is coupled with a withdrawal of the inhibitory effects of progesterone on the myometrium following a fall in plasma levels of this steroid. To determine whether maintenance of elevated circulating levels of progesterone during late pregnancy could positively modulate Hic-5 expression, pregnant rats were given either a daily injection of progesterone (4 mg, sc, in 0.2 mL corn oil), to maintain elevated plasma levels of this steroid, or vehicle alone (corn oil, 0.2 mL sc) beginning on d20 of gestation. This regime of progesterone administration resulted in delayed labour on d23. Individual animals were euthanized as described above and tissue collected as previously described [15].

### Tissue collection

Uterine horns were removed, opened longitudinally, and fetuses and placentas discarded. Tissue was placed in ice – cold phosphate – buffered saline (PBS; pH 7.4) and the endometrium was separated from the myometrium as described previously [14]. Myometrium samples were flash frozen in liquid nitrogen and stored at -80°C until used or fixed overnight in zinc buffered fixative [15,23] (ZBF; 100 mM Tris buffer pH 7.4, 3 mM calcium acetate, 27 mM zinc acetate, 37 mM zinc chloride) while shaking at room temperature. Tissues were embedded in paraffin, sectioned, and mounted on microscope slides by the Histology Unit of the Faculty of Medicine at Memorial University of Newfoundland.

#### Northern blot analysis

Northern blot analysis for the normal pregnancy regime was performed on four separate, independent sets of RNA samples (n = 4, i.e. 4 rats used per gestational timepoints) while analysis for the delayed labour regime was performed on three separate, independent sets of RNA samples (n = 3). RNA was isolated from tissues using TRIzol^® ^Reagent (Cat # 15596-018; Invitrogen Corporation, Burlington, Ontario, Canada) exactly according to the manufacturer's instructions. RNA samples were prepared for electrophoresis, electrophoretically separated and capillary transferred exactly according to White et al [24]. RNA was crosslinked to nylon membrane with a UVC-508 ultraviolet crosslinker (Ultra-Lum Inc., Paramount, California, USA) and all blots were stored at -20°C until required.

#### Northern blot hybridization

Northern blot hybridization procedures have been described in detail elsewhere [15,24]. Briefly, membranes were pre-hybridized in hybridization buffer then hybridized overnight at 42°C in hybridization buffer containing a ^32^P-labelled Hic-5 cDNA probe. The mouse Hic-5 cDNA template (Genbank Accession #L22482) was generously provided by Dr. Kiyoshi Nose (Showa University School of Pharmaceutical Sciences, Tokyo, Japan) and was found to have 94% identity with the corresponding rat cDNA (Genbank Accession #AF314960). Radiolabelled cDNA probes were prepared with a Megaprime DNA Labeling Kit according to the manufacturer's protocol (Cat # RPN 1607; Amersham Biosciences, Little Chalfont, Buckinghamshire, England). Following hybridization, blots were washed in 2 × SSC/0.1 % SDS at 42°C and then exposed to X – ray film (Hyperfilm MP; Amersham Biosciences). Multiple exposures were produced for each Northern blot to ensure the results were within the linear range of the film. Following analysis of Hic-5 gene expression, Northern blots were stripped in an aqueous solution of 1 M Tris-Cl, 1 mM EDTA, 0.1× Denhardt's then washed at room temperature with 0.1× SSPE. Northern blots were subsequently analyzed for expression of 18S rRNA using the same procedures described above and a rabbit 18S ribosomal cDNA template generously provided by Dr. I. Skerjanc (University of Ottawa, Ottawa, Ontario, Canada). 18S rRNA is constitutively expressed in rat myometrial cells and has been utilized, in the past, as a loading control for analysis of myometrial mRNA expression [7,15,24,25].

### Immunoblot analysis

Immunoblot analysis for both normal pregnancy and delayed labour regimes was performed on three separate, independent sets of protein samples (n = 3, i.e. 3 rats used per gestational timepoint) as previously described [14,15,24]. Frozen rat myometrial samples were pulverized under liquid nitrogen with a mortar and pestle and homogenized in RIPA lysis buffer [50 mM Tris-HCl (pH7.5), 150 mM NaCl, 1 % (vol/vol) Triton X-100, 1 % (wt/vol) sodium deoxycholate, 0.1 % (wt/vol) SDS] containing 100 μM Na_2 _VO_3 _and COMPLETE™ Mini EDTA-free protease inhibitors (Cat # 11617900; Roche Molecular Biochemicals, Laval, Quebec, Canada). Samples were centrifuged at 15,000 × *g *at 4°C for 15 min and the supernatants collected. Protein concentrations were determined by the Bradford Assay [26]. Protein samples (50 μg/lane) were separated by polyacrylamide gel electrophoresis in 9 % resolving gels and gels electroblotted to Pierce 0.45 μm nitrocellulose membrane (Pierce Biotechnology, Inc, Rockford, IL, USA).

All blot incubations were completed at room temperature with constant agitation unless otherwise stated. Membranes were rinsed in Tris buffered saline (20 mM Tris base, 137 mM NaCl, pH 7.6) with 0.1 % Tween-20 (TBST) then blocked in 5 % milk powder (5 % milk powder, 95 % TBST) for 40 min. A mouse monoclonal Hic-5 specific antibody (Cat # 611164; BDBiosciences, Mississauga, Ontario, Canada), diluted 1:2000 in blocking solution, was added to the blots and incubated for 1 hour. Blots were rinsed in TBST then a horseradish peroxidase-conjugated goat anti – mouse IgG (Cat # 31430; Pierce Biotechnology, Inc, Rockford, IL, USA) secondary antibody, diluted 1:20 000 in blocking solution, was added to the blots and incubated for additional 45 min. Immunoblots were then rinsed in TBST and proteins were detected using the Pierce SuperSignal West Pico Chemiluminescent Substrate detection system (Cat # 34080; Pierce Biotechnology, Inc, Rockford, IL, USA) and multiple exposures were generated to ensure the linearity of the film exposures. Following immunoblot analysis of Hic-5 expression, blots were stripped with Restore™ Western blot stripping solution according to the manufacturer's instructions (Cat # 21059; Pierce Biotechnology, Inc, Rockford, IL, USA). Subsequently, analysis of calponin protein expression (Antisera Cat # C2687; clone hCP; Sigma-Aldrich, Oakville, Ontario, Canada) was performed for use as a normalization control [15,24].

### Immunofluorescence

Two separate, independently collected sets of rat tissues (n = 2, i.e. 2 rats used per gestational timepoint) were utilized for immunofluorescence experiments and experiments were repeated three times. Five micrometer thick paraffin sections of myometrial tissue were placed on glass slides and dried overnight at 37°C. Two serially cut uterine tissue sections were present on each slide for experiments with one always serving as the negative control. Sections were dewaxed in xylene (3 × 5 min), rehydrated in descending grades of ethanol and soaked in PBS. Heat-induced epitope retrieval was accomplished using a solution of 0.01 M SSC, pH 6.0 as described by Williams et al. [15].

All subsequent steps were performed at room temperature unless otherwise noted. Tissue sections were blocked in 5 % normal goat serum, 1 % horse serum in PBS with agitation. Sections were then incubated for 1 hour in mouse monoclonal Hic-5-specific antisera (Cat # 611164; BDBiosciences, Mississauga, Ontario, Canada), diluted 1:50 with blocking solution, or mouse IgG (Cat # 015-000-003; Jackson Immunoresearch Labs Inc., West Grove, PA, USA) at the same concentration to serve as a negative control. Following washes in PBS, tissues sections were incubated in Rhodamine-Red-X conjugated donkey anti-mouse IgG (Cat # 715-295-150; Jackson Immunoresearch Labs Inc.), diluted 1:200 in blocking solution, with gentle agitation for 45 min. Sections were then washed with ice cold PBS containing 0.2 % Tween 20. For double immunofluorescence experiments assessing Hic-5/FAK co-localization, the above procedure was subsequently repeated utilizing rabbit polyclonal FAK-specific antisera (Cat #sc-558; Santa Cruz Biotechnology, Santa Cruz, CA, USA), diluted 1:50 in blocking solution, or rabbit IgG (Cat # 011-000-003; Jackson Immunoresearch Labs Inc.) at the same concentration to serve as a negative control. An FITC-conjugated sheep anti-rabbit IgG (Cat # F7512; Sigma, St. Louis, Missouri, USA), diluted 1:250 in blocking solution, was used as the secondary antisera.

All tissue sections were mounted in Vectashield mounting media (Vector Laboratories, Inc, Burlington, Ontario, Canada) and slides observed using an Olympus Fluoview 300 laser scanning confocal microscope (Olympus Optical Company Ltd., Melville, New York, USA) or a Leica DMIRE2 imaging system equipped with epi-fluorescence optics (Leica Microsystems, Richmond Hill, Ontario, Canada) and OpenLab™ image analysis software (Improvision Limited, Coventry, UK).

### Data analysis

Densitometric analysis of Northern blots and immunoblots were performed with the aid of Scion Image software (Scion Image Corporation, Frederick, Maryland, USA). Densitometric measurements of Hic-5 mRNA were normalized to those of 18S rRNA while measurements of Hic-5 protein on immunoblots were normalized to calponin. Statistical analysis was performed with GraphPad Instat version 3.0 (GraphPad Software, San Diego, California, USA) and data graphed using GraphPad Prism version 4.0 (GraphPad Software). Northern blot and immunoblot data during normal pregnancy were subjected to a one-way analysis of variance and unpaired Student t-tests. Northern blot data from delayed labour experiments were subjected to a two-way analysis of variance and Bonferroni post-tests. In all cases, values were considered significantly different if p < 0.05.

## Results

### Hic-5 mRNA and protein expression during pregnancy and labour

**Figure 1:**
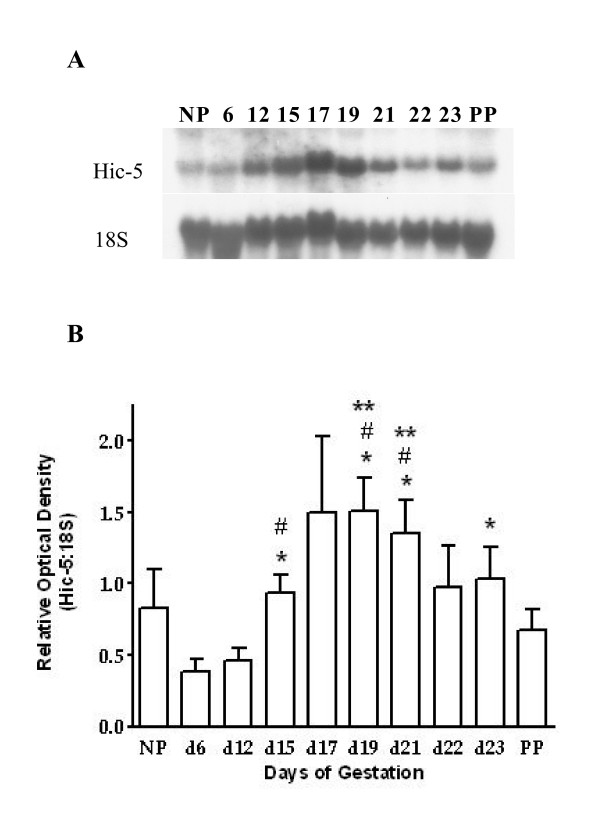
Northern blot analysis of Hic-5mRNA expression in rat myometrium during pregnancy, labour and 1 day postpartum. (A) Representative Northern blots of Hic-5 mRNA expression and 18S rRNA expression. Analysis was performed with a Hic-5-specific mouse cDNA and an 18S ribosomal rRNA-specific rabbit cDNA as templates for radiolabelled probe production. (B) Densitometric analysis illustrating the increase of Hic-5 mRNA expression during late pregnancy. Hic-5 mRNA expression on d15, d19, d21, and d23 (active labour) was significantly elevated compared to d6 (p < 0.05; *) and expression on d15, d19, and d21 was significantly elevated compared to d12 (p < 0.05; #). Furthermore, Hic-5 mRNA expression was significantly elevated on d19 and d21 (p < 0.05; **) compared to PP. Values are from 4 independent experiments (n = 4) ± SE. Days 6, 12, 15, 17, 19, 21, 22 and 23 represent gestational timepoints. NP = non-pregnant, PP = 1 day postpartum.

To evaluate Hic-5 mRNA expression throughout pregnancy and labour, myometrial tissue was collected from non – pregnant (NP) rats and from pregnant rats on gestational days (d) 6, 12, 15, 17, 19, 21, 22, 23 (active labour), and 1 day post – parturition (PP) for Northern blot analysis. Northern blots of rat myometrial total RNA were analyzed using radiolabelled probes created from a mouse Hic-5 specific cDNA template. Our experiments demonstrated that Hic-5 mRNA expression significantly increased during late pregnancy (one-way ANOVA, p < 0.05; Fig. 1). Specifically, Hic-5 mRNA expression on d15, d19 and d21 was significantly elevated compared to d6 and d12 of pregnancy and expression on d23 was significantly elevated over d6 (unpaired t – test, p < 0.05). Furthermore, Hic-5 mRNA expression was significantly elevated on d19 and d21 compared to PP (unpaired t – test, p < 0.05).

**Figure 2:**
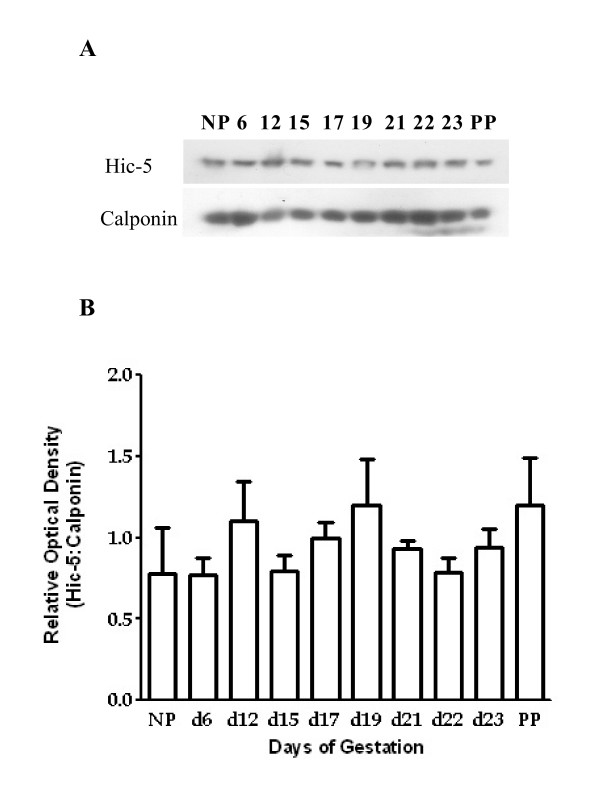
Immunoblot analysis of Hic-5 protein expression in rat myometrium during pregnancy, labour and postpartum. (A) Representative immunoblots of Hic-5 and calponin expression. (B) Densitometric analysis illustrating no overall statistically significant increases in Hic-5 detection in the rat myometrium throughout the time course examined. Values are from 3 independent experiments (n = 3) ± SE. Days 6, 12, 15, 17, 19, 21, 22 and 23 (active labour) represent gestational timepoints. NP = non-pregnant, PP = 1 day postpartum.

Immunoblot analysis, utilizing monoclonal Hic-5 specific antisera, demonstrated that Hic-5 protein was detectable in rat myometrial tissue lysates throughout pregnancy and in non-pregnant rat myometrial samples. Specifically, there were subtle fluctuations in Hic-5 detection at d12, d19, and PP but overall there were no statistically significant increases in Hic-5 detection in the rat myometrium throughout the time course examined (one-way ANOVA, p > 0.05; Fig. 2). Goat polyclonal Hic-5 specific antisera (Cat # sc-17616; Santa Cruz Biotechnology, Inc.) was also used for immunoblot analysis and gave similar results (data not shown).

**Figure 3:**
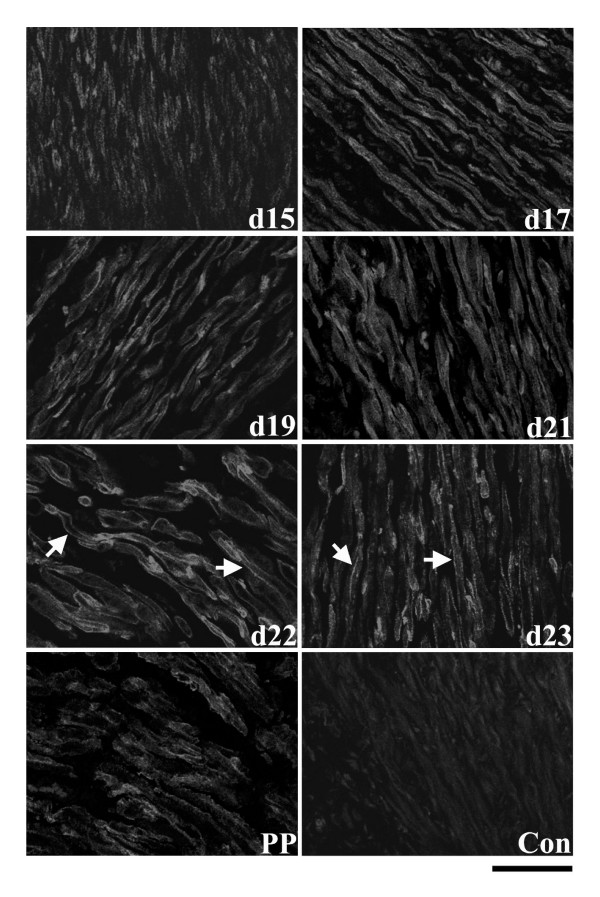
Immunolocalization of Hic-5 protein in the circular smooth muscle layer of rat myometrium at d15, d17, d19, d21, d22, d23 of gestation and post-partum (PP). Hic-5 was detectable in the cell cytoplasm and more continuously associated with myometrial cell membranes, particularly on d22 and d23 (arrows). Control = mouse IgG. Scale bar = 50 μm.

In the circular muscle layer, immunofluorescence experiments demonstrated that Hic-5 was not as readily detectable from NP to d15 of gestation compared to later timepoints. Spatially, Hic-5 was immunolocalized to both the cell cytoplasm and at cell membranes from NP to d15 (data not shown). In contrast, immunofluorescence detection of Hic-5 protein appeared to increase from day 17 onwards except PP when Hic-5 detection again appeared to diminish (Fig. 3). During these periods of gestation Hic-5 was detectable in the cell cytoplasm and more continuously associated with myometrial cell membranes, particularly on d22 and d23. At PP, cell membrane-associated staining became much more discontinuous throughout the tissue layer.

**Figure 4:**
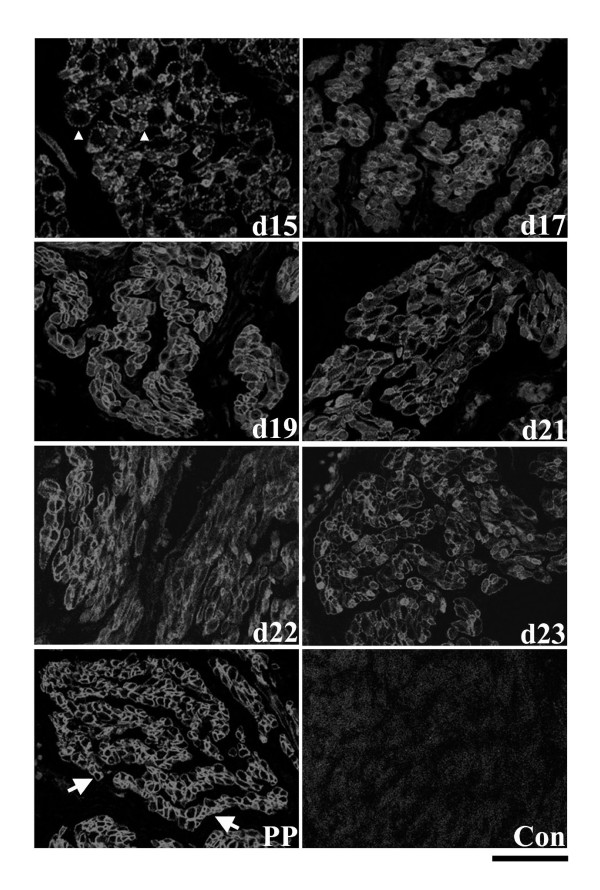
Immunolocalization of Hic-5 protein in the longitudinal smooth muscle layer of rat myometrium at d15, d17, d19, d21, d22, d23 of gestation and post-partum (PP). On d15 Hic-5 was detectable as distinct, well separated punctate foci at plasma membranes (arrowheads) while at subsequent timepoints, and particularly in PP samples, Hic-5 became consistently detected as an almost continuous line of intense fluorescence at cell membranes (arrows). Control = mouse IgG. Scale bar = 50 μm.

In contrast to the circular smooth muscle layer, Hic-5 was more readily detectable in the longitudinal muscle layer. From NP to d12, Hic-5 was immunolocalized at a low level in the cytoplasm of myometrial cells (data not shown). At all timepoints thereafter, Hic-5 was readily detectable and primarily associated at myometrial cell membranes (Fig. 4). Specifically on d15, Hic-5 was detectable as distinct, well separated punctate foci at plasma membranes while at subsequent timepoints, and particularly in PP samples, Hic-5 became consistently detected as an almost continuous line of intense fluorescence at cell membranes (Fig. 4).

A goat polyclonal Hic-5 specific antisera also tested for immunoblot analysis (above) did not give us any results, for comparison, in our immunofluorescence assays under our experimental conditions (data not shown). Furthermore, a rabbit polyclonal Hic-5 antisera (Cat # 4914, Cell Signaling Technology, Inc.) that was available to us was not used for immunofluorescence assays due to potential cross-reactivity with paxillin in situ.

### Hic-5 mRNA and protein expression during delayed labour

**Figure 5:**
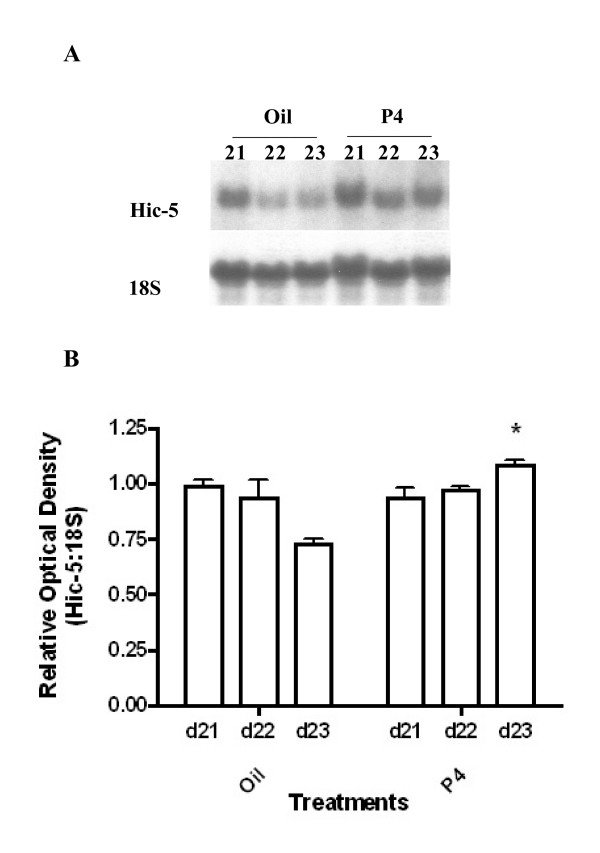
Northern blot analysis of Hic-5 mRNA expression following administration of progesterone or corn oil (vehicle control) to pregnant rats. (A) Representative Northern blots of Hic-5mRNA expression and 18S rRNA expression. (B) Densitometric analysis illustrating a significant increase (p < 0.05) in Hic-5 mRNA expression on d23-P4 compared to d23 vehicle controls (active labour). Values are from 3 independent experiments (n = 3) ± SE. P4 = progesterone. Designations 21-Oil, 22-Oil, 23-Oil (active labour), 21-P4, 22-P4, 23-P4 represent gestational time-points in the two treatment groups.

With our finding that Hic-5 mRNA expression increased significantly at d19 and d21, periods associated with high circulating levels of progesterone during rat pregnancy [27], we investigated whether maintenance of elevated circulating levels of progesterone in the rat from d21 – 23 might positively modulate Hic-5 mRNA expression. Northern blot analysis revealed that progesterone administration (thus delaying labour) resulted in a significant increase in Hic-5 mRNA expression (two-way ANOVA; p < 0.05; Fig. 5). Specifically, Hic-5 mRNA expression on d23-delayed labour in progesterone-treated rats was significantly higher compared to expression on d23-active labour in vehicle treated rats (Bonferroni post-test; p < 0.05).

**Figure 6:**
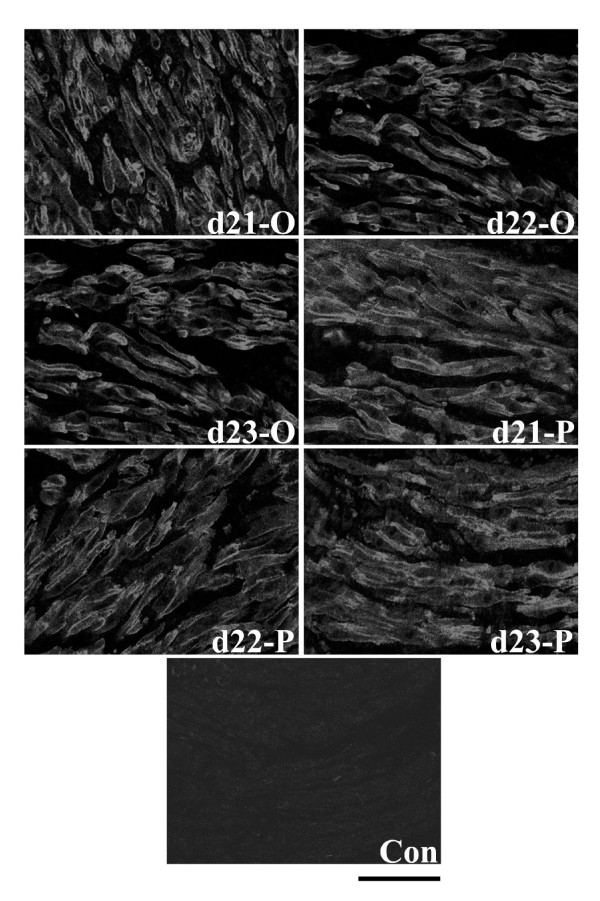
Immunofluorescence analysis of Hic-5 protein expression in the circular smooth muscle layer of rat myometrium following administration of progesterone (4 mg in 0.2 ml corn oil sc) or oil (vehicle control; 0.2 ml corn oil sc). There were no consistent, marked changes in detection of Hic-5 protein in myometrial tissue sections from progesterone-treated rats compared to vehicle controls. P = progesterone. O = oil. Con = mouse IgG. Days 21-O, 22-O, d23-O (active labour), 21-P, 22-P, and 23-P represent gestational time-points. Scale bar = 50 μm.

**Figure 7:**
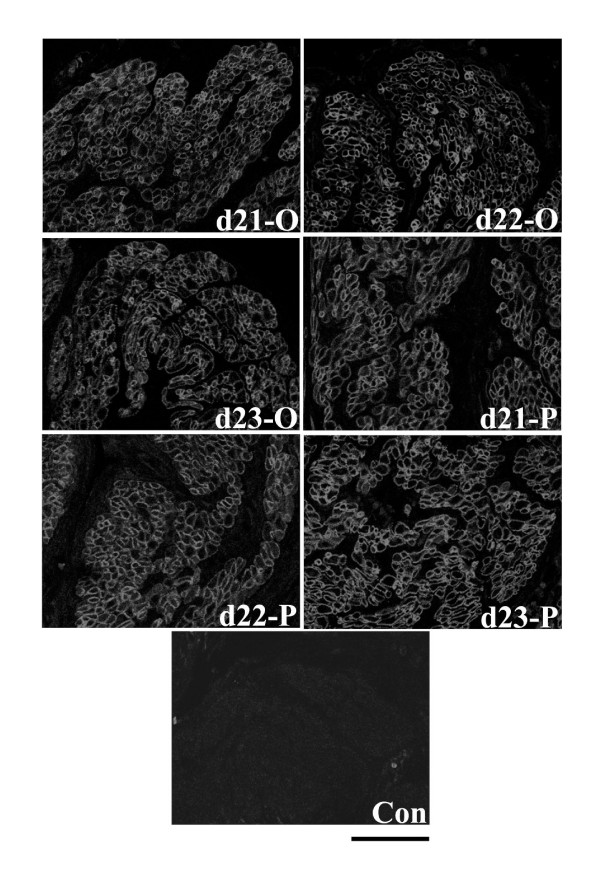
Immunofluorescence analysis of Hic-5 protein expression in the longitudinal smooth muscle layer of rat myometrium following administration of progesterone (4 mg in 0.2 ml corn oil sc) or oil (vehicle control; 0.2 ml corn oil sc). There were no consistent, marked changes in detection of Hic-5 protein in myometrial tissue sections from progesterone-treated rats compared to vehicle controls. P = progesterone. O = oil. Con = mouse IgG. Days 21-O, 22-O, d23-O (active labour), 21-P, 22-P, and 23-P represent gestational time-points. Scale bar = 50 μm.

Despite the change in Hic-5 mRNA expression upon progesterone administration, immunofluorescence experiments demonstrated that in the circular and longitudinal muscle layers there were no consistent, marked changes in detection of Hic-5 protein in myometrial tissue sections from progesterone-treated rats compared to vehicle controls. Specifically, in both muscle layers Hic-5 was readily and primarily detected associated with myometrial cell membranes with low levels of Hic-5 detected in the cytoplasm of cells within the circular muscle layer (Figs. 6, 7).

### Co-immunolocalization of Hic-5 and FAK in rat myometrium

**Figure 8:**
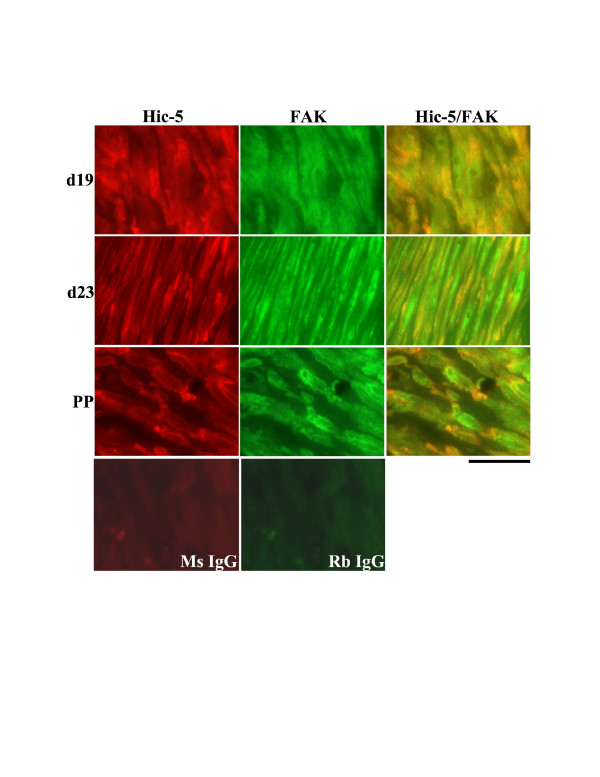
Co-immunolocalization analysis of Hic-5 and FAK in the circular smooth muscle layer of rat myometrium at d19, d23 (active labour) of gestation and post-partum (PP). A limited level of Hic-5/FAK association (yellow) was observed both in the cytoplasm and at membranes of myometrial cells. Ms IgG = mouse IgG negative control, Rb IgG = rabbit IgG negative control. Scale Bar = 50 μm.

**Figure 9:**
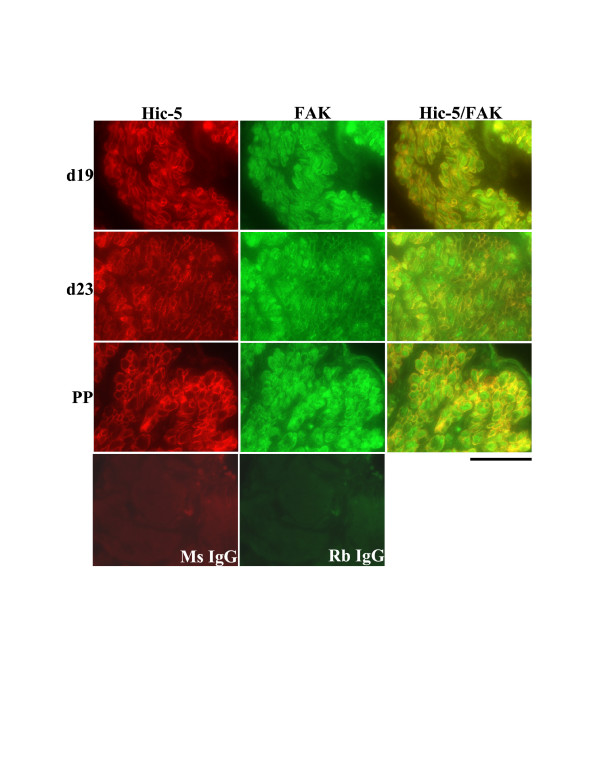
Co-immunolocalization analysis of Hic-5 and FAK in the longitudinal smooth muscle layer of rat myometrium at d19, d23 (active labour) of gestation and post-partum (PP). Hic-5 and FAK were readily co-localized at myometrial cell membranes (yellow) while there was very limited co-localization in the cytoplasm of myometrial cells where FAK was also readily detectable. Ms IgG = mouse IgG negative control, Rb IgG = rabbit IgG negative control. Scale Bar = 50 μm.

In the circular muscle layer of the rat myometrium on d19, d23 and PP, Hic-5 was detected in the cytoplasm of myometrial cells and associated with cell membranes. FAK was detected primarily in the cytoplasm of myometrial cells with a low level detected at the plasma membranes. Thus, a limited level of co-localization was demonstrated during late pregnancy, labour and PP in this muscle layer (Fig. 8). In contrast, in the longitudinal muscle layer during the same time points Hic-5 and FAK were readily co-localized at myometrial cell membranes while there was very limited co-localization in the cytoplasm of myometrial cells where FAK was also readily detectable (Fig. 9).

## Discussion

### Normal pregnancy and labour

Our Northern blot experiments demonstrated that Hic-5 mRNA expression increased significantly over gestation, particularly during the second half of gestation on d15, d19, d21 and labour. This timeframe of gestation is characterized by hypertrophic growth of the myometrium, induced by progesterone and uterine distension caused by growing fetuses, and follows a period of myometrial hyperplasia [4,5,9]. With the switch to hypertrophic myometrial growth, and associated ECM reorganization [7,9,10], focal adhesion remodeling must occur to support these processes and this remodeling can occur in a tension-dependent manner [14,28]. Thus, it is possible the increase in Hic-5 mRNA expression we observed may be induced by increased uterine stretch; however, at present there have been no reports of Hic-5 mRNA expression being regulated by mechanical forces. In relation to the observed increases in Hic-5 mRNA expression, in both smooth muscle layers of the myometrium Hic-5 protein became more readily detectable by immunofluorescence at ~d15 – d17 of gestation. This could be the result of translation of the increased levels of Hic-5 mRNA that were detected at approximately this time.

Of note, our immunoblot analysis did not demonstrate any statistically significant increases in Hic-5 detection in the rat myometrium throughout the time course examined, in contrast to Hic-5 mRNA expression. Since we found muscle layer-specific differences in Hic-5 expression by immunofluorescence analysis, we believe that a lack of significant detection differences in Hic-5 protein over gestation by immunoblot analysis may be due to "averaging" of Hic-5 protein levels within total protein lysates prepared from myometrial tissue samples containing both muscle layers.

Immunofluorescence experiments not only demonstrated that Hic-5 was more detectable in the longitudinal muscle layer than the circular layer of the myometrium, but that the spatial pattern of expression of Hic-5 differed at times between the two muscle layers. In the circular muscle layer, Hic-5 was detectable both in the cytoplasm and associated with membranes of myometrial cells while in the longitudinal muscle layer, particularly after d12, Hic-5 was detectable primarily at myometrial cell membranes. The functional consequences of these patterns of expression are unclear, but the two muscle layers do possess different embryological origins, contractile and physiological characteristics [29-32].

From mid-pregnancy onwards, circumferential uterine growth occurs in association with fetal growth and uterine tension is likely predominant in the circular muscle layer [9]. Thus, Hic-5 protein detection in the cytoplasm of myometrial cells of the circular layer may be a response, at least in part, to uterine stretch. Recently, Guignandon et al. [33] and Kim-Kaneyama et al. [21] have shown that Hic-5 protein expression can be responsive to mechanical stretching relocalizing from focal adhesions to the cytoskeleton in mouse embryonic fibroblasts, COS7 cells, Ros 17/2.8 osteoblast-like cells, and SVS30 smooth muscle cells. Our future work with a unilaterally pregnant rat model will more specifically determine the role of uterine stretch as a regulatory mechanism for both Hic-5 mRNA and protein expression in the myometrium. Interestingly, the transient over-expression of Hic-5 in SVS30 smooth muscle cells, embedded in 3D collagen gels, suppressed their contractile capability and this was dependent on Hic-5 association with the cytoskeleton [21]. Therefore, based on this work it may be possible that Hic-5 has a role in suppressing myometrial cell contraction, particularly in the circular muscle layer, from mid to late pregnancy.

During late pregnancy, labour, and PP, Hic-5 became more continuously associated at myometrial cell membranes; in the circular muscle on d22 and d23 and in the longitudinal muscle layer between d17 and PP. The over-expression of Hic-5 in fibroblasts is known to significantly reduce cell spreading on fibronectin and stimulate motility of murine mammary gland cells [20,34]. Both events are associated with prevention of stable focal adhesion formation, suggesting that in the myometrium during late pregnancy Hic-5 may be involved in focal adhesion remodeling which would correlate with the marked reorganization of the ECM in the rat uterus at this time [7,10]. A primary mechanism of Hic-5 regulation of cell spreading appears to involve competing for FAK binding with paxillin [20] and this may also occur in myometrial cells since both FAK and paxillin are expressed in the rat myometrium during late pregnancy [14].

### Effects of progesterone on Hic-5 expression

Throughout the majority of rat pregnancy circulating levels of progesterone are high, with levels of 25.7 ng/mL by d19 declining to 1.14 ± 0.52 ng/mL by d23 [27]. Since Hic-5 mRNA expression increased significantly at d19 and d21, periods associated with the high circulating levels of progesterone, we investigated whether maintenance of elevated circulating levels of progesterone in the rat from d21-23 might modulate Hic-5 mRNA expression. Indeed, administration of progesterone to pregnant rats significantly increased levels of Hic-5 mRNA on d23 (delayed labour) compared to d23 vehicle controls (active labour) suggesting progesterone may have a role in regulating Hic-5 mRNA expression. This role might be indirect, perhaps resulting from the prevention of labour contractions per se, or may be direct via transcriptional regulatory mechanisms. Since it was possible that an averaging of Hic-5 protein levels could be occurring in our immunoblot analysis, we directly examined Hic-5 protein detection and spatial localization in rat myometrial tissue in situ following administration of progesterone or vehicle alone. The lack of any marked changes in immunofluorescent detection of Hic-5 protein in myometrial tissue sections from progesterone-treated rats, compared to vehicle controls, suggests that the potential effects of progesterone on Hic-5 gene expression may only be manifested at the transcriptional level. We cannot rule out, however, that the discrepancy between Hic-5 mRNA and protein expression in our progesterone study could be due to a relatively longer half-life of Hic-5 protein relative to Hic-5 mRNA. In future experiments, we will administer the progesterone receptor antagonist RU486 (or vehicle) to pregnant rats to induce pre-term labour and more definitively determine if progesterone has a role in the regulation of Hic-5 gene expression in the myometrium.

### Co-immunofluorescent detection of FAK and Hic-5

FAK has previously been shown to be highly expressed and activated in the rat myometrium during late pregnancy, at a time when there is substantial remodeling of the ECM [10,14]. Hic-5 is reported to associate with FAK in epithelial cells and fibroblasts [14,18,20,35], but whether this association occurs in the myometrium is unknown. Thus, we examined the likelihood of Hic-5/FAK association by co-immunofluorescence analysis. Our experiments revealed that in the circular muscle layer there was a potentially limited level of Hic-5/FAK association both in the cytoplasm and at membranes of myometrial cells during late pregnancy, labour and post-partum. In contrast, there appears to be a potentially high level of association of Hic-5/FAK primarily at myometrial cell membranes of the longitudinal muscle layer. Thus, at least during these gestational timepoints in the rat myometrium there is a good probability of focal adhesion signaling involving a FAK-Hic-5 protein complex.

### The role(s) of Hic-5 during pregnancy

Scaffolding proteins, like Hic-5, contain modular protein-binding domains that allow them to mediate many protein-protein interactions simultaneously, thus acting as hubs for the integration of a variety of signaling pathways [36,37]. We did not detect Hic-5 in cell nuclei in either smooth muscle layer under our experimental conditions. Hic-5 can shuttle between focal adhesions and the nucleus and several reports indicate that Hic-5 can participate in the transcriptional regulation of the c-fos gene, as a scaffold in transcriptional complexes, and can also act as a steroid receptor coactivator [38-41]. We cannot completely rule out such roles for Hic-5 in the myometrium during pregnancy, but our results at this time suggest that its primary role in the myometrium during late pregnancy may lie close to or at the myometrial cell membrane as a focal adhesion scaffolding protein. The LIM and LD domains in the Hic-5 molecule which can mediate protein-protein interactions would support this task and Hic-5 is reported to bind several focal adhesion proteins such as Hsp27, vinculin, paxillin, and FAK [17,18,20,42-44]. All four of these latter proteins have also been readily detected in the rat myometrium during late pregnancy [14,24] and our co-localization experiments have indicated that there is a good probability of focal adhesion signaling involving a FAK-Hic-5 protein complex during late pregnancy. Since focal adhesions function as cytoskeletal attachment sites and likely assist in the transduction of force across the muscle to create a mechanical syncytium [16], modulation of FAK/paxillin complexing by Hic-5 may be an important signaling mechanism for development of such a mechanical syncytium during late pregnancy prior to the onset of labour. Thus, this study serves as the foundation for more specific analyses of the role of Hic-5 in FAK/Paxillin complexing, focal adhesion remodeling, and myometrial cell contraction.

## Conclusion

Hic-5 is highly expressed in the rat myometrium during late pregnancy and labour and co-localizes with FAK in situ. Our results are consistent with a potential role for Hic-5 in focal adhesion remodeling in the rat myometrium during late pregnancy.

## Authors' contributions

JMC carried out the Northern blot analysis, immunoblot analysis, the bulk of the immunofluorescence experiments, and performed the statistical analyses. LRGP assisted with a small number of immunofluorescence experiments. DJM conceived and designed the study, assisted with statistical analyses and drafted the manuscript. All authors read and approved the final manuscript.
